# OCT Biomarkers in a Cohort of Patients With *PRPF31*-Associated Retinitis Pigmentosa

**DOI:** 10.1155/joph/6629368

**Published:** 2025-09-07

**Authors:** Jan-Philipp Bodenbender, Katarina Stingl, Susanne Kohl, Laura Kühlewein

**Affiliations:** ^1^University Eye Hospital, Centre for Ophthalmology, University of Tübingen, Tübingen, Germany; ^2^Institute for Ophthalmic Research, Centre for Ophthalmology, University of Tübingen, Tübingen, Germany

**Keywords:** ellipsoid zone, inherited retinal dystrophy, optical coherence tomography, *PRPF31*, retinitis pigmentosa

## Abstract

**Purpose:** With degeneration of the photoreceptors in retinitis pigmentosa (RP), the reflectivity of the ellipsoid zone (EZ) decreases. We aimed to study characteristics of the EZ and its reflectivity in a cohort of patients with *PRPF31*-associated autosomal-dominant RP (*PRPF31*-adRP) and a possible relationship to EZ width and best-corrected visual acuity (BCVA).

**Methods:** EZ width and relative EZ reflectivity (rEZR) were assessed in 32 patients with *PRPF31*-adRP. EZ width was measured on a horizontal SD-OCT scan through the fovea of the right eye. On the same OCT scans, rEZR was measured at the foveola, temporal and nasal parafoveola and fovea.

**Results:** Total EZ width revealed a significant negative correlation with age (*r*_*s*_ = −0.418, *p*=0.017). Foveolar rEZR revealed a significant negative correlation with age (*r*_*s*_ = −0.403, *p*=0.022), but was not significantly correlated with BCVA (foveolar: *r*_*s*_ = −0.151, *p*=0.410), in contrast to parafoveolar rEZR (*r*_*s*_ = −0.365, *p*=0.0399), which was significantly correlated with BCVA. The ratio of foveal and parafoveolar rEZR correlated significantly with total EZ width (*r*_*s*_ = 0.358, *p*=0.044).

**Conclusions:** The EZ reflectivity (EZR) can be measured reliably using freely available software. The correlation we observed between rEZR and BCVA leads us to the conclusion that rEZR may be an additional marker in observational and therapeutic trials.

## 1. Introduction

Retinitis pigmentosa (RP) is the most common inherited retinal dystrophy [1, 2]. It is characterized by a progressive degeneration of rods, subsequently followed by cones, and typically starting in the mid-periphery [3–5]. RP is caused by over 3000 mutations in more than 70 different genes [1, 2]. Variants in the *PRPF31* gene (pre-mRNA processing factor 31, OMIM ∗606419) are the second most common cause of autosomal dominant RP (RP11, OMIM #600138), accounting for up to 10% of cases [6–11]. In our local cohort, *PRPF31*-adRP accounts for 3% of IRD, 6% of RP, and ∼20% of ADRP cases [12, 13]. Recently, a phase 1 clinical trial for RNA therapy was launched (clinicaltrials.gov NCT05902962), to evaluate the safety and tolerability of intravitreally administered VP-001 in participants with confirmed *PRPF31*-associated retinal dystrophy.

Objective measurement of photoreceptor degeneration is important for monitoring disease and outcome in therapeutic clinical trials [6, 14, 15]. Functional measurements include best-corrected visual acuity (BCVA), visual field (VF), and electrophysiology. Structural measurements include spectral domain optical coherence tomography (SD-OCT) and fundus autofluorescence (FAF) imaging, which are well-correlated [16]. Functional deficits precede structural changes [16]. In the VP-001 study, secondary outcome measures included, among others, BCVA, kinetic perimetry, retinal thickness on SD-OCT, ellipsoid zone (EZ) area and volume, and area of hypoautofluorescence captured by FAF.

In OCT, the EZ width reflects photoreceptor function [17]. Its measurement reveals a low test-retest variability, and so changes in EZ width reach statistical significance faster than those observed in electroretinography (ERG) or VF testing [18]. With degeneration of the photoreceptors, EZ reflectivity (EZR) decreases, making it a potential biomarker for detection of photoreceptor degeneration in RP [4, 15, 19–21].

The aim of this study was to determine the characteristics of EZR in a cohort of patients with *PRPF31*-associated RP and their possible relationship to BCVA, age, and EZ width.

## 2. Methods

### 2.1. Study Design

The study was conducted in accordance with the principles of the Declaration of Helsinki and approved by the Ethics Board of the Medical Faculty of the University of Tuebingen (study nos. 310/2022BO2, 116/2015BO2, and 349/2003V). We included patients from our tertiary referral center for hereditary retinal degenerations with heterozygous pathogenic variants in the *PRPF31* gene. Written informed consent was on hand from all patients (or their parents or legal guardians).

### 2.2. Patients

All patients with a disease-associated variant in *PRPF31* who presented to our hereditary retinal disease consultation since 2013 and who provided informed consent were included in the study. Patients were excluded from measurements if they revealed a posterior staphyloma on OCT imaging, if the EZ line was centrally interrupted, if the EZ line extended beyond the field of view, or if the EZ width was less than 500 μm temporal or nasal from the foveola, making a foveal reflectivity measurement impossible.

### 2.3. Ophthalmological Assessments

The ophthalmological examination included BCVA using a numeric eye chart measuring decimal visual acuity that was converted to the logarithm of the minimum angle of resolution (logMAR) [22]. A horizontal SD-OCT scan through the fovea of the right eye was used for measuring EZ width and reflectivity. The scans were captured by the same OCT device (Spectralis HRA + OCT, Heidelberg Engineering GmbH, Heidelberg, Germany), the same 30° standard lens, and the same acquisition mode (non-EDI) within the clinical routine.

### 2.4. Outcome Measures

**Figure 1:**
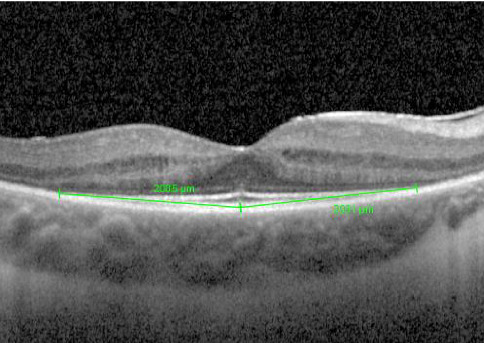
EZ width was measured by determining the endpoints of the EZ, measuring from each endpoint to the EZ line at the foveola, and adding these two distances.

EZ width was measured by determining the endpoints of the EZ according to the specifications of Ramachandran et al. [14]. Measurements were taken from each endpoint to the EZ line at the foveola and these two distances were added (Figure 1). Measurements for each scan were done independently by two physicians using the distance tool of the Heidelberg Eye Explorer (HEYEX).

**Figure 2:**
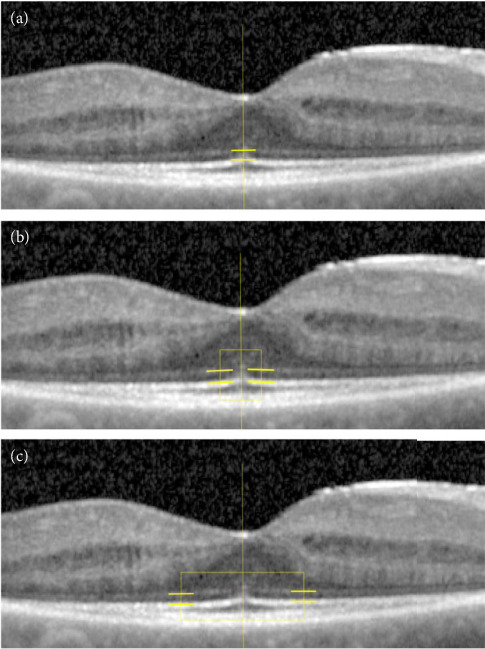
The reflectivity of the ellipsoid zone and the external limiting membrane was measured at the foveal centre (foveola, (a)), at the foveolar ring (parafoveola, (b)) and at the foveal ring (fovea, (c)), each over a width of 200 μm.

For measuring the EZR, logarithmic displayed B-scans (tagged image file format (TIFF), 8 bits per pixel, 256 different gray values) were imported into ImageJ (National Institutes of Health, Bethesda, MD). Average reflectivity (mean gray value) of the EZ and the external limiting membrane (ELM) was obtained from five regions of interest (ROI), each 17 pixels (approximately 200 μm) wide and one pixel high, localized at the foveal centre (here referred to as foveola), at the foveolar ring (here referred to as parafoveola, 14 pixels, approximately 150 μm temporally and nasally of the foveola), and at the foveal ring (here referred to as fovea, 44 pixels, approximately 500 μm temporally and nasally of the foveola) (Figure 2). The mean pixel intensity of each ROI was calculated. The reflectivity of the EZ line was normalized by calculating the relative EZR (rEZR) as the ratio of the mean EZR to the mean ELM reflectivity at the same location, to account for patient- and image-specific differences such as media opacities, as has been done in comparable studies investigating EZR [21, 23–25]. The ratio of the foveal and the foveolar rEZR and the ratio of the foveal and the parafoveolar rEZR, as well as the ratio of the parafoveolar and the foveolar rEZR, were calculated to relate the reflectivity of more peripheral parts of the retina to central reflectivity, as central reflectivity most likely represents the original rEZR before photoreceptor damage, since in RP photoreceptor damage progresses towards the center of the retina. The measurements of EZR were repeated for 20% of the patients (*n* = 7, randomly selected) by the same technical staff member.

### 2.5. Statistical Analysis

The Wilcoxon matched-pairs signed-rank test was used to calculate potential differences between rEZR at different locations. Spearman's rank correlation test was used to determine the correlation between age, BCVA, rEZR, rEZR ratios, and EZ width. For quantitative assessments of intergrader agreement in EZ-line measurement and intragrader agreement in EZ-reflectivity measurement, intraclass correlation coefficients (ICC, two-way mixed, absolute agreement) with 95% confidence intervals (CI) were calculated. A *p* value of less than 0.05 was considered statistically significant. A correlation coefficient lower than 0.30 was considered weak, a correlation coefficient between 0.30 and 0.50 moderate, and a correlation coefficient larger than 0.50 strong [26]. Data analysis was performed with SPSS (IBM SPSS Statistics for Windows, Version 28.0.0.0, IBM Corp., Armonk, New York, USA).

## 3. Results

### 3.1. Patient Profile

In total, data from 86 patients with *PRPF31*-associated adRP were available. Two patients were excluded due to a posterior staphyloma, 19 due to a central disruption of the EZ line, four due to the EZ line extending beyond the OCT scan area, 23 because the EZ radius was less than 500 μm, and six patients were excluded due to the absence of OCT data. Thirty-two patients were included in the study, of which 78.13% (25/32) were female. The median age of the patients was 34 years (mean 37.28 years, range, 14–74 years). Median BCVA [logMAR] was 0.15 (mean 0.34, range 0.00–2.70).

### 3.2. EZ Width

**Figure 3:**
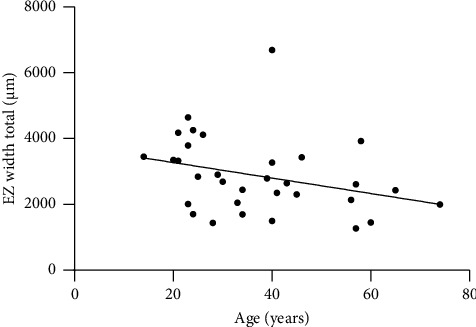
Total horizontal EZ width of patients with *PRPF31*-associated retinitis pigmentosa. Note the decline in EZ width with increasing age.

The mean total EZ width was 2865 μm, ranging from 1272 to 6689 μm. Mean temporal EZ width was 1477 μm (range, 643–3620 μm), and mean nasal EZ width was 1388 μm (range, 629–3069 μm), correlating statistically significantly (*r*_*s*_ = 0.908, *p* < 0.001). The independent measurements by both physicians were highly correlated with an ICC of 0.989 (0.656–0.997). Total EZ width revealed a statistically significant moderate correlation with age (*r*_*s*_ = −0.418, *p*=0.017) (Figure 3). There was no statistically significant correlation between total EZ width and BCVA (*r*_*s*_ = −0.3480, *p*=0.051).

### 3.3. EZ Reflectivity

**Figure 4:**
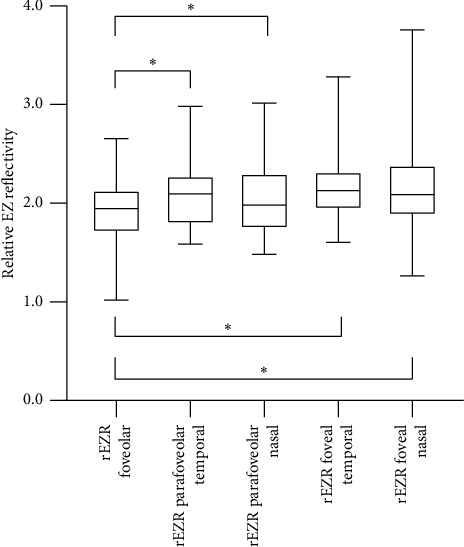
Relative ellipsoid zone reflectivity (rEZR) in patients with *PRPF31*-associated retinitis pigmentosa obtained at different regions of interest: foveola, temporal and nasal parafoveola, and temporal and nasal fovea. Note that rEZR is smallest in the foveola.

**Figure 5:**
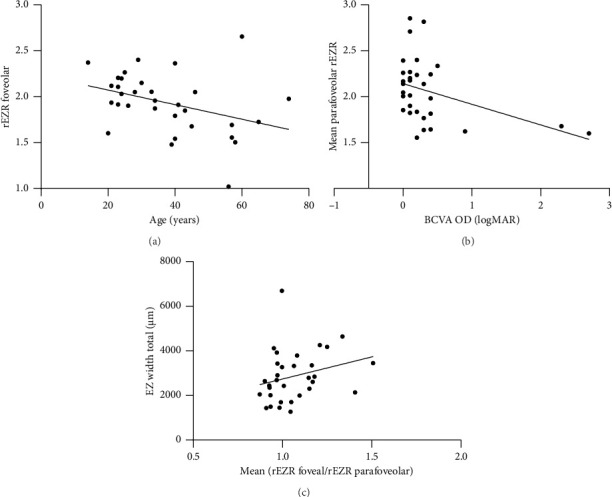
(a) Foveolar relative ellipsoid zone reflectivity (rEZR) in patients with *PRPF31*-associated retinitis pigmentosa plotted against age. Note the decline in foveolar rEZR with increasing age. (b) Mean parafoveolar rEZR plotted against best-corrected visual acuity (BCVA). Note the decline in BCVA with decreasing mean parafoveolar rEZR. (c) Mean of the ratio of the foveal and parafoveolar rEZR plotted against the EZ width. Note the decline in EZ width with decreasing ratio.

Mean foveolar rEZR was 1.934 (range 1.019–2.655), temporal parafoveolar rEZR 2.093 (range 1.583–2.982), nasal parafoveolar rEZR 2.038 (range 1.481–3.016), temporal foveal rEZR 2.175 (range 1.603–3.282), and nasal foveal rEZR 2.180 (range 1.264–3.757) (Figure 4). The foveolar rEZR was significantly lower than the parafoveolar and foveal rEZR, whereas there was no statistically significant difference between the latter as well as between the temporal and nasal side at both locations. The repeated measurements of the rEZR revealed a high correlation for all locations, although there were outliers in the foveolar measurements (foveola: ICC 0.927 (0.158–0.989), temporal parafoveola: ICC 0.967 (0.832–0.994), nasal parafoveola: ICC 0.953 (0.754–0.992), temporal fovea: ICC 0.972 (0.855–0.995), nasal fovea: ICC 0.994 (0.966–0.999)). Statistically significant correlations were seen between foveolar rEZR and age (*r*_*s*_ = −0.403, *p* = 0.022), between the mean parafoveolar rEZR and BCVA (*r*_*s*_ = −0.365, *p* = 0.040), and between the ratio of the foveal and parafoveolar rEZR and total EZ width (*r*_*s*_ = 0.358, *p* = 0.044) (Table 1, Figure 5, Supporting Figure S1–S7).

## 4. Discussion

Here, we studied characteristics of the EZ line in a comparably large and genetically homogeneous cohort of patients with *PRPF31*-adRP.

### 4.1. EZ Width

EZ width displayed a considerably large range even though patients with EZ widths below 1000 μm were excluded. EZ width correlated significantly with age despite a notably wide variance when comparing patients of similar age. This is in line with variable expressivity and reduced penetrance of *PRPF31*-adRP and is likely due to the proposed pathomechanism in *PRPF31*-adRP [27]. It is assumed that there is a threshold value below which the disease manifests itself depending not only on the loss of protein (function) of the mutant allele but also on the expression level of the wild-type counter allele [28, 29]. It is suggested that the level of expression determines the severity of the disease, leading to significant intra- and interfamilial differences [28], which is in accordance with our findings. EZ width did not correlate with BCVA which met our expectations as BCVA is driven by the integrity and functionality of the central retinal tissue and patients with advanced disease, i.e., central retinal involvement, were excluded from our analysis.

### 4.2. EZ Reflectivity

The photoreceptor degeneration in RP is concentric with a transition zone between the demised and vital photoreceptors, where photoreceptors are still present but already damaged [4]. In this area, EZR is reduced [4, 21], the photoreceptor layer is thinned, and the thickness of the inner and outer segments is diminished [4]. This reflectivity difference along the EZ was shown in our study, without any significant difference between temporal and nasal sections of the retina, which is in line with a concentric affection of photoreceptors. The median rEZR was close to 2.00 in all measured locations, reflecting a two-fold higher reflectivity of the EZ compared to the ELM on average in our cohort of PRPF31-adRP patients. This is in line with the results of Wu et al., investigating the rEZR within 1000 μm of the foveola in AMD patients. Wu et al. reported a significantly lower rEZR in subjects with early AMD (1.77 ± 0.26) compared to healthy control participants (1.95 ± 0.27, *p* < 0.001) [25]. In our cohort, the foveolar rEZR was significantly lower than the parafoveolar and foveal rEZR, a result, which was also reported in other studies [25, 30]. This might be caused by a different packing density of the mitochondria in the cones at the foveola and further peripherally. The cones at the foveola are described as long and thin, whereas the cones further peripherally are shorter and wider, resulting in an increased packing density of the mitochondria in the vertical direction [25].

The foveolar rEZR correlated significantly with age, which has also been reported by Wu et al. in AMD patients [25]. We can only speculate whether this decline in rEZR is attributable to ageing or, in the case of our cohort, RP disease progression, or both. This would need to be verified in larger studies including healthy subjects and ideally more RP patients.

The foveolar rEZR did not correlate significantly with BCVA; in contrast, mean parafoveolar rEZR revealed a statistically significant correlation with BCVA. Gong et al. showed a correlation of foveolar rEZR with BCVA in 22 eyes of 22 RP patients [4]. However, Gong et al. calculated the average relative optical intensity of the EZ by dividing the peak of the EZ by the mean reflection of the whole retina at the same position within the central 1 mm of the retina. Therefore, the measurement is less compartmentalized, covering also the parafovea up to the fovea on both sides, so that rEZR in these sections of the retina might also here be responsible for the correlation. Sousa et al. also found a correlation between the presence of a subfoveal EZ and BCVA in RP patients [3]. However, Sousa et al. only distinguished between the presence and absence of the foveolar EZ line. Both studies included patients in advanced stages of the disease and did not exclude patients with a diminished EZ line in the fovea, i.e., shorter than 500 μm temporal or nasal to the foveola. Based on the findings from our study, follow-up studies could be carried out in the future, including patients in more advanced stages of RP, i.e., EZ lengths of 250 μm only temporally and nasally of the foveola, respectively.

The ratio of foveal and parafoveolar rEZR correlated statistically significantly with EZ width. We deem the parafoveola better suited for rEZR measurements and so for acting as a reference point that represents the original rEZR, as it correlates with BCVA and the EZ line is straight at the parafoveola in contrast to the tilt of the EZ line in the foveola which may alter the reflectivity there. The tilt of the EZ line in the foveola is also likely to be the cause for a lower intergrader agreement when measuring the reflectivity in the foveola compared to other locations.

When comparing results between different studies on EZR, one must consider that there are different approaches for measuring EZR. In addition to the manual delineation, there is the possibility of the determination of a local maximum value using reflectivity profiles [21, 23–25, 30]. Since we chose manual delineation, we could ensure that the local maximum values of the ELM and EZ were selected within the layers. Reflectivity profiles are especially relevant for an automatic determination. For each ROI a range of 200 μm was measured at each of the different locations, and an average value was calculated. This is in line with other studies [23–25, 30]. A width of 200 μm allows to average over several pixels while still allowing an adaptation to a slight curvature of the retina. The ROI was adjusted according to any observed curvature of the retina, which in some cases proved to be challenging in the foveola, where the EZ line is typically elevated. Normalizing EZR is necessary to account for differences between image sections and patients, however, there is no standardized method [21]. We chose to measure the rEZR as the ratio of the reflectivity of the EZ line to the reflectivity of the ELM, similar to other studies [23–25]. Others have determined the reflectivity in relation to the retinal pigment epithelium [31, 32] or to the total reflectivity of the outer retina [19, 30]. The ELM was chosen here, as it represents a non-neural layer, its reflection is thought to originate from the zonulae adhaerentes between the photoreceptors and Müller cells [25]. Either TIFF images [23, 25] or raw data [24] can be used for reflectivity analysis. In TIFF images, OCT data are transformed and logarithmically represented, according to the use in clinical practice for facilitating distinctions in hyporeflective ranges. Raw data images represent the actual differences in reflectivity. However, working with raw data requires extensive computing, making it almost impossible to use in clinical practice.

### 4.3. Strengths and Weaknesses of the Study

Our study has several strengths. We established a method with low retest variability to quantitatively assess the rEZR, applying widely used software rather than complex research software. Additionally, we were able to show that the measurement was possible using OCT scans made in clinical routine. Another strength of the study is that all measurements were performed on a rather large cohort of RP patients with variants in the same gene.

Our study also has limitations. Firstly, in comparable studies, ELM was chosen as a reference when measuring the rEZR, as it represents a non-neural layer and reveals a consistent intensity across the retina in AMD [23–25]. The situation is different with inherited retinal dystrophies, where there is also a loss of the ELM. However, as this occurs later than EZ loss, in our opinion ELM can be used for measurements. Secondly, for phenotypes that reveal no well-defined retinal layers since early childhood (e.g., CRB1), such a reflectivity measurement is not possible. Thirdly, we only included patients in early or intermediate stages of RP, and our findings likely do not apply to patients with more advanced disease. Lastly, we measured the rEZR at fixed locations rather than measuring it in the respective individual transition zones. Measuring the peripheral reflectivity at an individually adapted distance from the foveola, adjusted to the EZ width, and thus in the transition zone, might provide even more insight than a measurement at a predefined distance that is the same for all patients.

## 5. Conclusions

Measuring the rEZR in RP patients with early or intermediate *PRPF31*-associated RP is possible with freely available software, using OCT scans made in clinical routine. Foveolar rEZR correlated statistically significantly with age and parafoveolar rEZR correlated statistically significantly with BCVA for *PRPF31*-adRP patients. Additionally, the ratio of foveal and parafoveolar rEZR revealed a statistically significant correlation with EZ width. rEZR offers additional information that may provide understanding of the natural course of disease and the effects of therapeutic measures in clinical trials. Besides, as the measurements are carried out at fixed distances from the foveola, it is much easier to shift the task to nonmedical technical staff members than it is with the measurement of the EZ width.

## Figures and Tables

**Table 1:** Correlation of EZ line reflectivity with age, BCVA, and EZ width.

	Variable	Correlation coefficient *r*_*s*_	*p* value
Foveolar rEZR	Age	−0.403	0.022^∗^
Foveolar rEZR	BCVA	−0.151	0.410
Mean parafoveolar rEZR	BCVA	0.365	0.040^∗^
Mean foveal rEZR	BCVA	0.267	0.139
Foveolar rEZR	EZ width	−0.085	0.642
Mean parafoveolar rEZR	EZ width	−0.111	0.546
Mean foveal rEZR	EZ width	0.128	0.485
Ratio of parafoveolar and foveolar rEZR	EZ width	−0.023	0.899
Ratio of foveal and foveolar rEZR	EZ width	0.301	0.094
Ratio of foveal and parafoveolar rEZR	EZ width	0.358	0.044^∗^

Abbreviations: BCVA = best-corrected visual acuity, EZ = ellipsoid zone, rEZR = relative ellipsoid zone reflectivity.

^∗^Statistically significant correlation.

## Data Availability

The data that support the findings of this study are available from the corresponding author upon reasonable request. The data are not publicly available due to privacy or ethical restrictions.

## Supporting Information

Supporting Figure S1: Foveolar relative ellipsoid zone reflectivity (rEZR) plotted against best-corrected visual acuity (BCVA).

Supporting Figure S2: Mean foveal rEZR plotted against BCVA.

Supporting Figure S3: Foveolar rEZR plotted against EZ width.

Supporting Figure S4: Mean parafoveolar rEZR plotted against EZ width.

Supporting Figure S5: Mean foveal rEZR plotted against EZ width.

Supporting Figure S6: Ratio of parafoveolar and foveolar rEZR plotted against EZ width.

Supporting Figure S7: Ratio of foveal and foveolar rEZR plotted against EZ width.
