# Supplementary material for: OCT Biomarkers in a Cohort of Patients With PRPF31-Associated Retinitis Pigmentosa
Source: J Ophthalmol. 2025 Sep 7;2025:6629368. doi: 10.1155/joph/6629368 (PMC12433729; doi:10.1155/joph/6629368)
Supplement: Supporting Information — Additional supporting information can be found online in the Supporting Information section. [file 6629368.f1.docx]

**OCT Biomarkers in a Cohort of Patients with PRPF31-associated Retinitis Pigmentosa**

*Jan-Philipp Bodenbender ^1^, Katarina Stingl ^1^, Susanne Kohl ^2^, Laura Kühlewein ^1^*

**Supplementary Material**


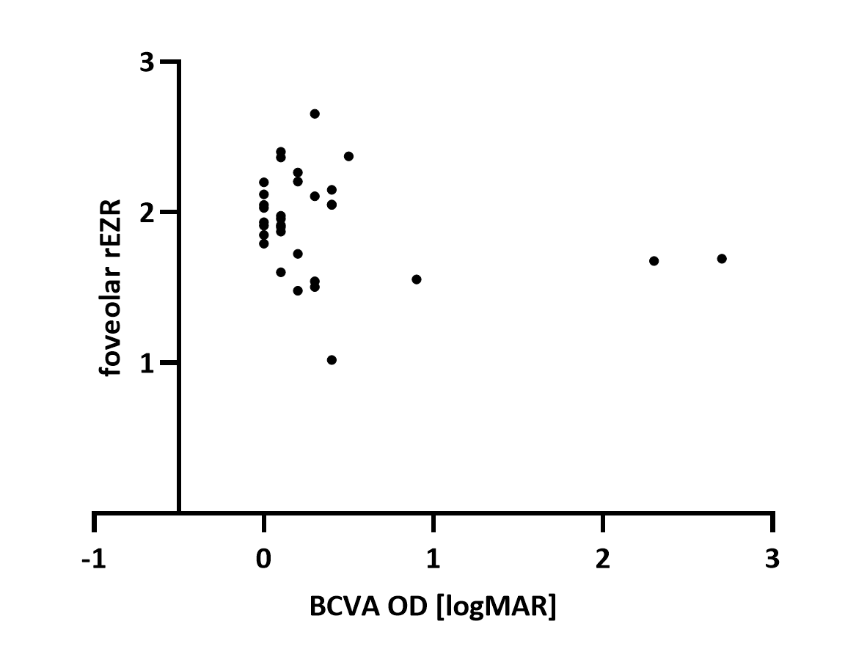


**Supplementary Figure S1:** Foveolar relative ellipsoid zone reflectivity (rEZR) plotted against best-corrected visual acuity (BCVA).


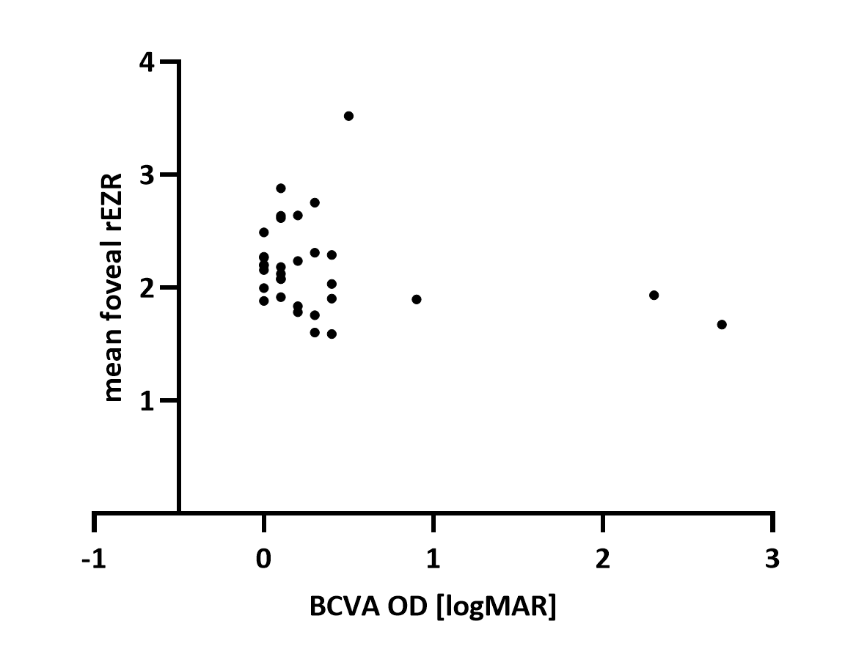


**Supplementary Figure S2:** Mean foveal rEZR plotted against BCVA.


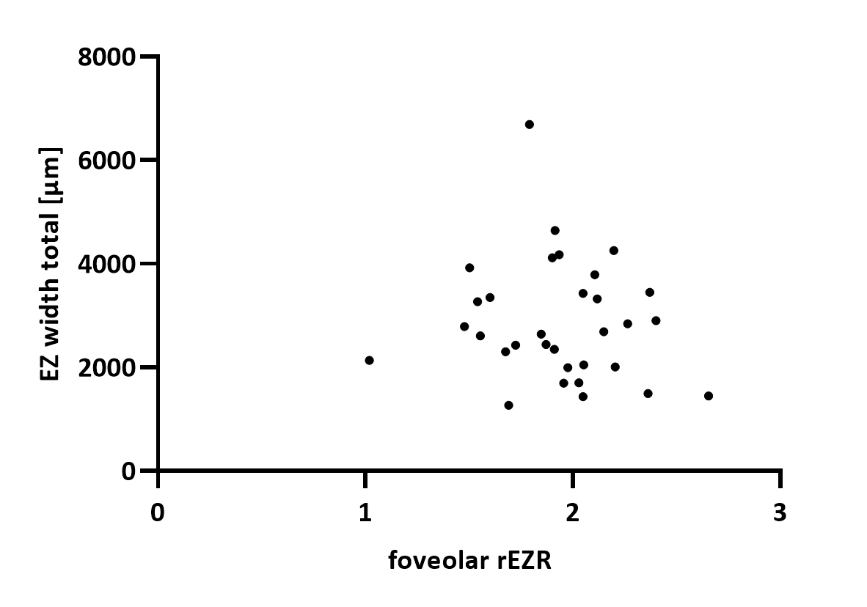


**Supplementary Figure S3:** Foveolar rEZR plotted against EZ width.


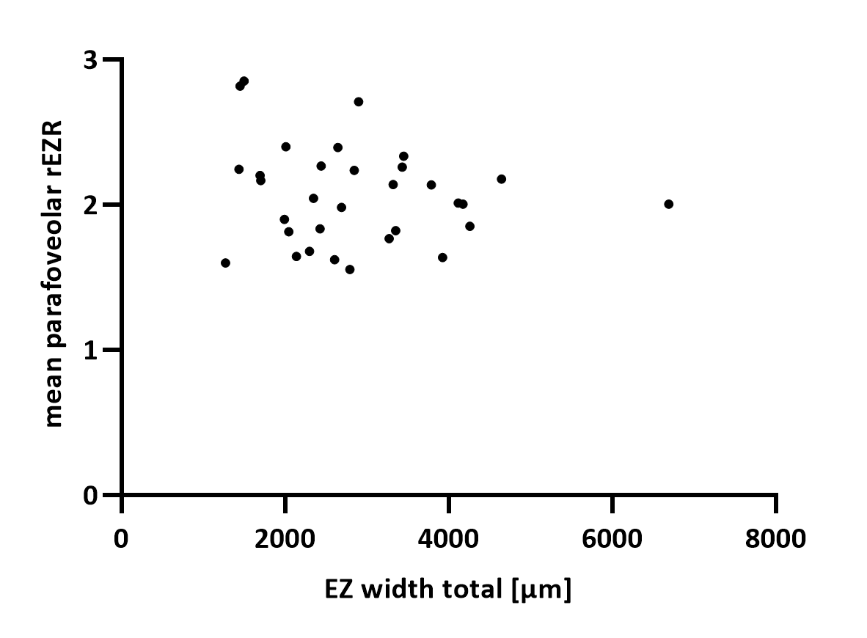


**Supplementary Figure S4:** Mean parafoveolar rEZR plotted against EZ width.


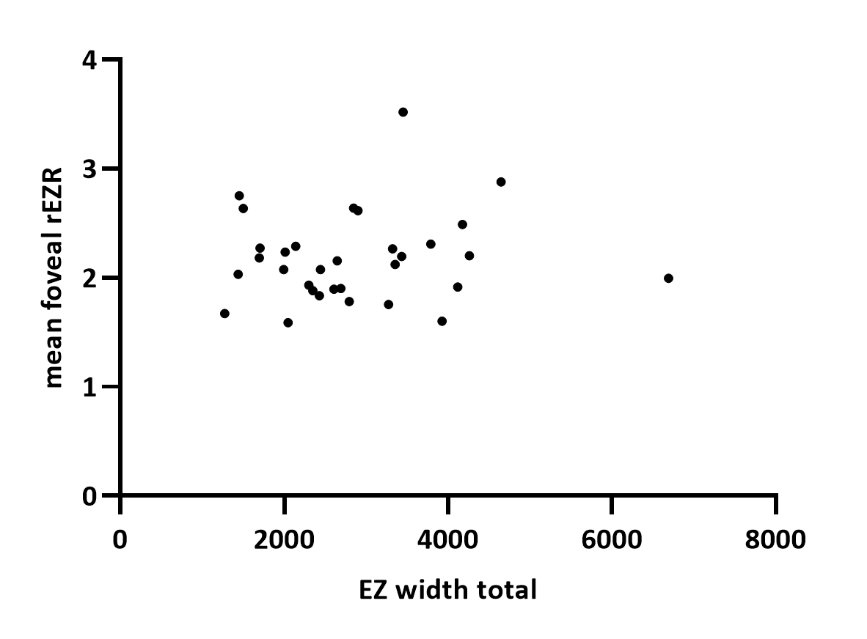


**Supplementary Figure S5:** Mean foveal rEZR plotted against EZ width.


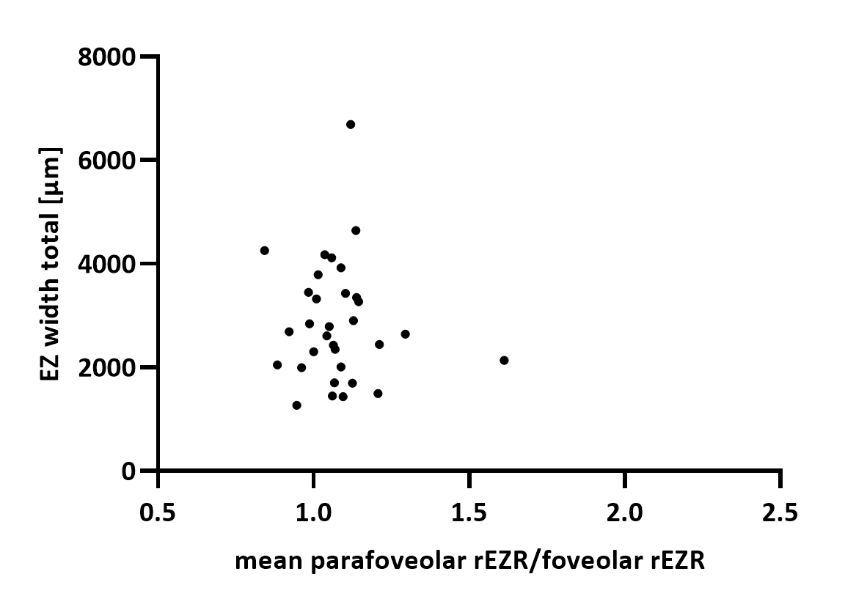


**Supplementary Figure S6:** Ratio of parafoveolar and foveolar rEZR plotted against EZ width.


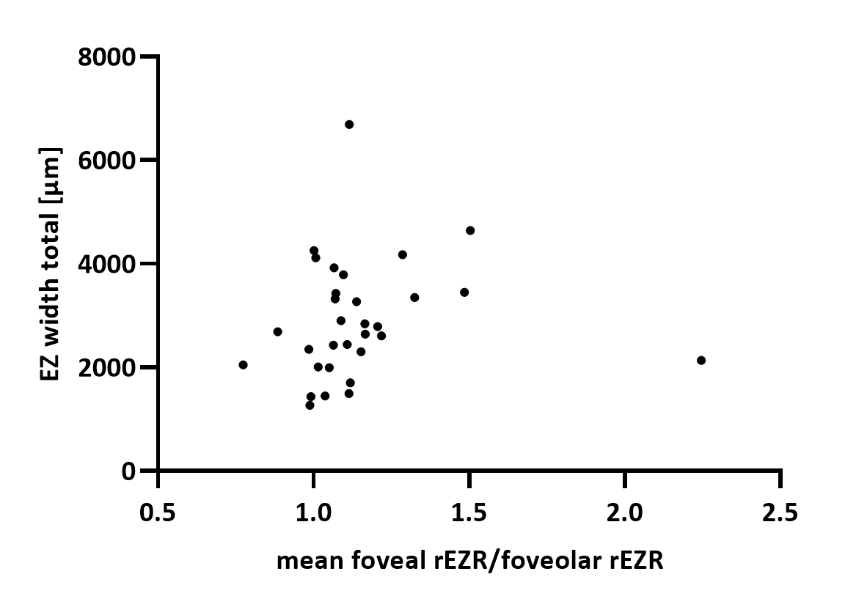


**Supplementary Figure S7:** Ratio of foveal and foveolar rEZR plotted against EZ width.
